# Hypertension and the Well-Being of African Migrants in South Africa

**DOI:** 10.3390/ijerph22050779

**Published:** 2025-05-14

**Authors:** Ufuoma Patience Ejoke, Edwin Devon Du Plessis

**Affiliations:** Department of Psychology, Faculty of the Humanities, University of the Free State, Bloemfontein P.O. Box 339, South Africa

**Keywords:** acculturation, African migrants, hypertension, public health, risk factors, South Africa

## Abstract

This article examines the risk factors for hypertension among migrants in South Africa, a critical public health concern. We explore the connection between acculturation, lifestyle changes, obesity, diet, urbanization, and socioeconomic status in contributing to hypertension risk. Our analysis highlights the unique challenges faced by African migrants, including acculturative stress, limited healthcare access, and lifestyle changes. The findings have significant implications for health promotion, disease prevention, and policy development. We emphasize the need for targeted interventions and updated immigration policies prioritizing hypertension awareness, screening, and management among African migrant populations.

## 1. Introduction

Although migration is accepted as a route to economic well-being in many communities and countries [1,2], the massive migration of people over the last 100 years has posed dynamics for several societies around the world and thrown entire communities into new, often stressful circumstances [3,4]. For most migrants, the adjustment process to life as an immigrant can be a stressful experience as they struggle to navigate major lifestyle changes, including language, economic, and social conditions [5]. These changes expose migrants to several dangers such as human trafficking, racial discrimination, exploitation, sexual abuse, brain drain, and loss of identity [6]. This burden of traumatic experiences compromises migrants’ sense of emotional well-being [5,7].

Well-being encompasses various aspects of positive functioning and overall quality of life, considering the context and circumstances that influence an individual’s experience [8]. In the communities where African migrants reside, access to health services, support, and care plays a crucial role in promoting positive functioning and evaluating a well-lived life.

Global migration is a complex issue driven by a multitude of factors, including health, economic, social, and security concerns, which are increasingly interconnected [9]. Notably, migrants comprise over 3.6% of the global population (IOM, 2022) [9]; their unique histories and experiences contribute to differences in health status between immigrant and native populations [2]. As migrants settle in new environments, they undergo significant lifestyle changes that can impact their health.

The transition from rural, agricultural environments to urban, industrial settings brings about significant changes in migrants’ lifestyles, including increased income, education, and alterations in family dynamics, dietary habits, and physical activity levels [10]. These changes can predispose individuals to various health conditions such as hypertension [11]. Moreover, migrants often encounter xenophobia-related barriers that hinder their access to healthcare services, ultimately compromising the management of chronic conditions like hypertension [12].

Hypertension, a leading non-communicable disease (NCD), significantly contributes to global mortality and morbidity [13]. According to WHO and UN-Habitat (2016) [13], NCDs are the new urban epidemic and have become one of the most pressing health policy issues [13]. NCDs disproportionately affect low- and middle-income countries (LMICs), with 80% of mortality occurring in these regions [14]. Sub-Saharan Africa bears a disproportionately high burden of NCDs, surpassing the global average, with hypertension being particularly prevalent in this region [15]. Notably, the burden of NCDs in Sub-Saharan Africa is now nearly equivalent to the combined burden of communicable, maternal, neonatal, and nutritional (CMNN) diseases [16].

The significance of hypertension cannot be overstated, as it is a leading cause of disability and death worldwide, contributing to cardiovascular disease, strokes, and kidney failure [17,18]. In South Africa, the demographic characteristics of migrants reveal that female migrants are more hypertensive than males due to the lack of awareness about behavioral risk factors [19]. Additionally, older migrants are more likely to be hypertensive, as they tend to neglect their health with age [19]. South Africa, hosting the largest immigrant population in Africa [20], provides a unique context in which to investigate hypertension and well-being among African migrants.

Eighty-six percent % of the migrant population living in South Africa is hypertensive [12]. An earlier migration study found that migration status is associated with the prevalence of hypertension. The authors argued that migrants are heterogeneous both in their origin status and migration histories [21]. In addition, migrants are vulnerable to hypertension because they lack awareness of the disease and have reduced access to healthcare [1,22]. For example, Mutambara and Naidu [23] found that Zimbabwean migrant women have challenges in accessing services from public hospitals and clinics in South Africa due to the lack of valid immigration documentation. Likewise, African migrants struggle to access healthcare services in their host countries [22].

Unfortunately, the lack of access or inadequate hypertension diagnosis and control in diagnosed patients increases morbidity and mortality. The latter places a strain on healthcare resources [24]. Managing these consequences is a challenge in Sub-Saharan Africa, where resource-intensive care is not readily available [25]. 

Hypertension and its associated mental health issues are overlooked conditions in the migrant population [12]. Furthermore, the need for more efforts in screening and connecting migrants’ needs to the health system has been recognized [1,12]. As such, the purpose of this article is to highlight risk factors for hypertension among the migrant population in order to inform the public health interventions required to adapt to changing healthcare practices and the health needs of African migrants.

## 2. South Africa and Migrants’ Health: An Overview

Migration, or the movement of people across borders [26], has been linked to issues of integration, adaptation, and health. Whether “voluntary” or “involuntary”, African migrants’ adaptation processes can be challenging as they strive to adjust to fundamental lifestyle changes such as language, and socioeconomic situations [12,27,28]. Many “involuntary” immigrants (refugees and asylum seekers) are burdened with traumatic experiences that can jeopardize their emotional well-being [5].

Most migrants live and work in deplorable conditions, thus exacerbating physical and mental health problems [11,28]. Such problems may have started in their home country or during their migration experience. Torture, abuse, and the loss of loved ones are all possibilities. As a result, a disproportionate percentage of immigrants, particularly young people, suffer from mood disorders, posttraumatic stress disorder (PTSD), anxiety, depression, and chronic pain [29,30,31]. Even in countries where illegal migrants have full access to healthcare, several obstacles prevent them from seeing a doctor. Language problems, a lack of understanding of, and access to, the health-care system, prohibitive charges, and the fear of deportation are among these [11].

South Africa has the highest number of immigrants on the African continent due to its middle-income position, strong democratic institutions, and comparatively industrialized economy. Official estimates place the country’s immigrant population at 2.9 million, or a little less than 5% of the country’s total population of 60 million people [32]. However, because of the enormous number of unlawful migrants, particularly from surrounding countries, the official estimates are regarded to be underestimates [20].

Several African countries are represented among the immigrants in South Africa, including Angola, Botswana, the Democratic Republic of Congo, Eswatini, Kenya, Malawi, Mozambique, Namibia, Nigeria, Somalia, Sudan, Tanzania, Zambia, and Zimbabwe [33]. Most South African immigrants originate from Botswana, Eswatini, Lesotho, Mozambique, and Zimbabwe, which are all neighboring nations. The rising demand for mine work contributed to an increase in the number of immigrants in the mid-1980s. The Renamo War in Mozambique in the 1990s resulted in an inflow of migrants into South Africa; these migrants are now frequently considered refugees. Kenya, Nigeria, and Zimbabwe account for the majority of work visa holders. Somalia has a large population of asylum seekers.

In recent decades, immigration has tended to rise, notably with the establishment of democracy at the end of Apartheid in 1994. Statistics South Africa, the government’s statistical office, estimates that a net 853,000 people migrated to the country between 2016 and 2021, a slight decrease from the net immigration of 916,300 in 2011–2016 but a significant increase from the 491,700 in 2001–2006. Net immigration was largest among African (894,400) and Asian (49,900) groups between 2016 and 2021 but was offset by the net emigration of roughly 91,000 White inhabitants. Most immigrants live in Gauteng, the country’s wealthiest province, which includes the commercial city Johannesburg, Pretoria, also known as the executive capital Tshwane, and the manufacturing hub of Ekurhuleni.

South Africa’s immigrant population is predominantly comprised of individuals from other African countries. According to the 2011 national census, approximately three-quarters of immigrants in South Africa originate from elsewhere on the continent. Notably, about 68% of these African immigrants come from outside the Southern African Development Community (SADC) region, which comprises 16 countries. Furthermore, United Nations data from 2020 reveals that Zimbabwe is the largest source country, accounting for nearly a quarter (24%) of all immigrants in South Africa. Immigrants from Europe and North America also flock to South Africa in large numbers. South Africa’s 2022 census reported approximately 2.4 million international migrants, making up 3.9% of the total population, a decrease from 4.2% in 2011. Remarkably, the peak age range for male migrants shifted from 25–29 to 35–39 years. However, census data may not accurately reflect recent migration trends due to its snapshot nature, potentially missing temporary or circular migrants who may not be present on the reference date [34].

The migration–health continuum has attracted academic debate. African migrants in LMICs are at greater risk of NCDs [12,18,35]. Cardiovascular risk factors are becoming more widespread in LMICs, particularly in South Africa, with a striking hypertension prevalence [36], and higher estimates of hypertension compared to other LMICs. However, hypertension is generally poorly characterized and controlled in South Africa due to insufficient medical equipment and manpower [37]. The incidence and effects of NCDs are projected to rise as treatment and care for other communicable diseases, such as HIV, improve and the epidemiologic transition continues. Hypertension is a leading cause of stroke and myocardial infarction, which can be fatal for both patients and the resource-constrained health systems that provide their care [18].

A study conducted by the South African National Health and Nutrition Examination Survey (SANHANES) found hypertension to be prevalent in 30.4% of the population, with significant geographical and demographic heterogeneity and rising prevalence as people become older [38]. Their findings also indicated how the screening and care for the high prevalence of HIV/AIDS interact with other diseases, notably non-communicable diseases, in South Africa. In the Agincourt sub-district of South Africa, hypertension was found to affect 43% of the population aged 35 and above. Hypertension was associated with both physical and social factors and was found in 84% of stroke survivors. Although only a few persons received treatment for hypertension, some were able to obtain good levels of control. Unreliable medicine supply and unreliable blood pressure measurement equipment were among the obstacles to providing successful treatment [39]. The study concluded that more diet information is needed. In rural South Africa, the awareness rate of hypertension was found to be 64.4%, treatment among those who were aware was 89.3%, and 45.8% of those treated were controlled. However, an interesting finding was that immigrants had lower levels of awareness, thus reflecting the vulnerability of the immigrant population [1]. 

Recent studies have highlighted the unique health challenges faced by migrants in South Africa, including a higher prevalence of NCDs such as hypertension, diabetes, and obesity. For instance, a study by Ajaero et al. [40] found that migrants in South Africa had a higher prevalence of NCDs (19.81%) compared to non-migrants (16.69%). The study, which analyzed data from the National Income Dynamics Study (NIDS), also revealed that older migrants, non-Black migrants, and those with higher education levels were more likely to have NCDs.

Internal migration, in particular, has been linked to an increased risk of NCDs, with migrant women being disproportionately affected. Pheiffer’s [18] study, which used panel data from the South African National Income Dynamics Study, found that internal migration was associated with higher blood pressure among women, but not among men. Similarly, a study by Pheiffer et al. [41] found that migrant women living in urban areas had higher levels of blood pressure compared to their rural counterparts.

Furthermore, migrants in South Africa often face significant barriers to accessing healthcare services, including medical xenophobia and social phobia. Akokuwebe et al.’s [42] study, which analyzed data from the Gauteng City-Region Observatory (GCRO) survey, found that migrant youths in Gauteng province faced significant challenges in accessing healthcare services, including medical exclusion and dissatisfaction with healthcare services.

The Migrant Health Follow-Up Study (MHFUS) has provided valuable insights into the health outcomes of internal migrants in South Africa. For example, a study by Ginsburg et al. [43] found that migrants had lower levels of depressive symptoms compared to non-migrants, but that migration status had a significant influence on depressive symptoms.

A study conducted by Munezero and Tomita [12] in Durban explored the mental health determinants of hypertension among refugees. The findings revealed a significant association between hypertension (blood pressure ≥ 130/90 mm Hg) and mental health challenges, particularly adverse childhood experiences. The study sampled 178 adult female African refugees/migrants seeking help; the results showed that 86% (*n* = 153) of the participants suffered from both depression and hypertension, the majority of participants were hypertensive, and depression was found to be independently associated with hypertension, regardless of smoking, alcohol consumption, and obesity. Their findings highlight the need for increased screening measures, because hypertension and related mental health issues often go undiagnosed among migrant populations.

Screening measures for infectious diseases, such as TB and several sexually transmitted diseases, are ensured for migrants and refugees to the USA and Europe, especially at the time of their application to adjust to permanent residence [44]. This focus on infectious diseases is also mirrored in the South African health screening system. Migrants present radiological and medical reports during permanent residence applications as a process for resettlement in South Africa, but not on chronic diseases such as hypertension [18], there are abundant data on migrants’ initial health status, their current health conditions after they have been living in their new home country after (re)settlement remain unknown [44], particularly their risk factors for hypertension.

## 3. Methods

### 3.1. Study Selection Criteria

We conducted a comprehensive literature review of peer-reviewed articles published in English between 2008 and 2024. We searched multiple databases, including PubMed, Scopus, and Web of Science, using keywords such as “hypertension”, “African migrants”, “South Africa”, “acculturation”, and “lifestyle changes”. We included studies that examined the relationship between acculturation, lifestyle changes, and hypertension risk among African migrants in South Africa.

### 3.2. Inclusion Criteria

Studies were included if:

1. They were published in English;
2. They were conducted among African migrant populations in South Africa;
3. They examined the relationship between acculturation, lifestyle changes, and hypertension risk;
4. They were published between 2008 and 2024.

### 3.3. Potential Biases

We acknowledge that our review may be subject to several biases. First, our search was limited to English-language articles, which may have excluded relevant studies published in other languages. Second, our focus on peer-reviewed articles may have excluded grey literature or unpublished studies that could have provided valuable insights. Finally, with a few included studies in the 1990s, our review was limited to studies conducted between 2008 and 2024, which may not capture the full scope of research on this topic.

## 4. Risk Factors for Hypertension

The underlying risk factors that contribute to hypertension can provide insight into why some people are more likely to acquire hypertension than others. Risk factors might be hereditary, behavioral, or environmental, or they can be the result of a medical condition. They can be treatable, irreversible, or linked to other predisposing conditions [45,46]. The World Health Organization [26] reports that non-communicable diseases tend to be of long duration and are the result of a combination of genetic, physiological, environmental, and behavioral factors. According to Agyemang [47], after adjusting for socioeconomic position, significant differences in the prevalence of hypertension between people of African and European heritage are considerably diminished. Environmental and lifestyle factors, rather than genetically determined ethnic differences, were found to be the main causes of hypertension.

Lurbe and Redon [2] explain that immigrants’ lifestyle and healthcare habits may be defined by habits formed in their native country, which they may progressively abandon as they adopt the host nation’s traditions. As their stay lengthens, this process may become more intensive. Stress (as a result of the process of acculturation), a lack of physical exercise, and dietary changes are the most prevalent factors that harm health. Acculturation is the process through which a person abandons the characteristics of a prior culture and adopts the characteristics of the prevailing culture in which he or she now lives. Economic difficulty, language, and cultural barriers, prejudice, and the loss of social, familial, and support networks are all linked to acculturative stress [48]. As a result of acculturation, the health of immigrants corresponds with that of the receiving country over time [49].

Hypertension is a well-known risk factor for cardiometabolic disease, which can be evaluated and treated with anti-hypertensive medication in primary healthcare systems at a low cost and with ease. From a public health standpoint, understanding the factors of increased blood pressure is an important aspect of treating the NCD problem in LMICs, both structurally and individually. Section 4.1, Section 4.2, Section 4.3, and Section 4.4 examine common risk factors for developing hypertension.

### 4.1. Obesity and Sedentary Lifestyle

Obesity and a sedentary lifestyle are well-known causes of hypertension [50]. Obesity-related hypertension associations have emerged as a serious public health concern around the world [51]. Complex mechanisms, such as altered hemodynamics, poor sodium homeostasis, renal dysfunction, autonomic nervous system imbalance, endocrine abnormalities, oxidative stress and inflammation, and vascular injury, are thought to mediate this association [52].

Migration can profoundly impact the development of obesity and sedentary lifestyles. As migrant populations adapt to new environments, they often undergo significant changes in their lifestyles, including reduced physical activity and changes in eating habits [53]. The transition from traditional, low-sodium, low-fat diets to processed foods and high amounts of salt can lead to an increased risk of obesity and related health problems. Furthermore, the stress of acculturation and adaptation to a new culture can also contribute to the development of obesity and a sedentary lifestyle. Research has shown that migrant populations, particularly the elderly, are more susceptible to these changes, which can have severe consequences on their health and well-being (Rosenthal, 2014) [53].

These findings suggest that obesity and sedentary lifestyles may be significant health concerns for African migrants in South Africa. As migrants adapt to a South African environment, they may be at an increased risk of developing unhealthy lifestyle habits, which can have long-term consequences for their health and well-being. In a study conducted in South Africa, migrant women in the Agincourt Health and Socio-Demographic Surveillance Site were disproportionately affected by obesity and sedentary lifestyles. Research by Ginsburg et al. [54] found that female migrants who resided outside the Agincourt study site throughout the four study waves were 1.8 times more likely to be overweight or obese than their non-migrant counterparts. Additionally, migrant women had significantly higher diastolic blood pressure levels, with an average of 4.3-mmHg higher diastolic BP compared to that of non-migrant women. These health disparities persisted over time and were not observed among male migrants. Migrant women may face unique health challenges that are not adequately addressed by the current healthcare system. A recent migration study found that migrant women were more likely to experience health disadvantages, including overweight, obesity, and higher BP levels, compared to non-migrant women [54].

A study conducted among Ghanaian migrants and their homeland counterparts found that high levels of C-reactive protein (CRP), a marker of inflammation, were associated with hypertension in urban Ghanaian women and European–Ghanaian men and women [55]. However, this association was largely explained by conventional risk factors, particularly body mass index (BMI).

According to Baleta and Mitchell [56], inadequate exercise and poor diets are the causes of the inevitable rise in obesity and its accompanying disorders; excessive alcohol intake and cigarette use are the other two major risk factors. Abukhdeir et al. [57] found that some lifestyle behaviors, such as smoking, are linked to hypertension and are a known risk factor for cardiovascular disease. Although there is no direct link between chronic smoking and blood pressure, smoking-induced arterial stiffness and wave reflection may have negative effects on central blood pressure, which is more directly linked to targeting organ damage than brachial blood pressure [58].

### 4.2. Diet

Diet is a significant risk factor for hypertension among African migrants in South Africa. As they transition from rural to urban environments, their eating habits change, often leading to increased consumption of processed and high-sodium foods [10]. This dietary shift can result in weight gain, a known independent risk factor for hypertension.

Research has shown that African migrants in South Africa tend to adopt a more Westernized diet, characterized by high levels of processed foods, sugar, and saturated fats [59]. This dietary pattern is associated with an increased risk of hypertension, particularly among migrant women [60]. A study found that African migrants in South Africa consumed more alcohol and fast food and had higher systolic and diastolic blood pressure compared to their counterparts [41].

The high salt intake among African migrants in South Africa is a significant concern. Research has shown that South Africans, including African migrants, consume significantly more salt than the recommended daily allowance [61,62]. The increasing salt intake can be attributed to the adoption of a Westernized diet, which often includes high-sodium processed foods.

Furthermore, the traditional diets of African migrants often undergo significant changes as they adapt to their new environment. For example, a study found that African migrants in South Africa tended to consume less traditional foods, such as leafy greens and whole grains, and more processed and high-calorie foods [63]. This shift away from traditional diets can lead to an increased risk of hypertension and other cardiovascular diseases.

Food insecurity and limited access to healthy food options can also contribute to the high prevalence of hypertension among African migrants in South Africa. Studies have shown that migrant populations often face barriers to accessing healthy food options, including limited financial resources, a lack of transportation, and the limited availability of healthy food options in their neighborhoods [64].

### 4.3. Urbanization

Urbanization is a significant factor contributing to the increasing burden of NCDs, particularly hypertension, in LMICs. According to the World Health Organization [26], 85% of the 15 million premature NCD-related deaths occur in LMICs each year, with elevated blood pressure (BP) being the leading metabolic risk factor for NCDs.

The relationship between migration, urbanization, and hypertension is complex and influenced by various social and behavioral factors. Economically vulnerable populations, including migrants, are disproportionately affected by NCDs [41]. The processes of migration and urbanization can exacerbate the risk of hypertension among migrant women, particularly in LMICs like South Africa. In Cameroon, migration to urban areas is linked to a higher body mass index (BMI), fasting blood sugar, and blood pressure [65]. BMI, a potent predictor of hypertension, is also substantially linked to urbanization and could be caused by dietary changes, decreased physical activity, increased psychological stress, and the disruption of traditional family ties.

Urbanization can have a disproportionate impact on the health of female migrants, particularly in low- and middle-income countries (LMICs). As women migrate to urban areas, they may experience greater isolation due to their employment opportunities and face increased safety risks compared to their male counterparts [13]. This can lead to elevated blood pressure and poor health outcomes [41].

The relationship between migration and blood pressure is likely influenced by psychosocial stressors, which are shaped by gendered migration systems and urban environments. As women adapt to urban life, they may encounter new stressors, such as discrimination, violence, and socioeconomic inequality, which can contribute to increased blood pressure and other health problems.

The Migrant Health Follow-Up Study (MHFUS) provides valuable insights into the relationship between internal migration and elevated BP among migrant women in South Africa [41]. The study’s findings highlight the importance of considering the social and behavioral factors that contribute to the migration–BP relationship, including household composition, social support, migration experience, and housing quality. Migrants also live in significantly smaller (1.4 members) households than non-migrants (4.6 members) on average. In the context of African migrants in South Africa, understanding the relationship between urbanization, migration, and hypertension is crucial for developing effective interventions to promote well-being and reduce the risk of NCDs.

### 4.4. Socioeconomic Status (SES)

Closely related to urbanization is socioeconomic status (SES). Educational and socioeconomic factors have been linked to hypertension awareness [66,67,68] and initiatives to connect edu-socio-economic factors more effectively to the health system should be considered. Better education and income were independently related to higher diastolic blood pressure in men, according to Cois and Ehrlich [69], who studied the socioeconomic determinants of hypertension in South Africa. Higher education was linked to lower diastolic and systolic blood pressure in women, while higher income was linked to lower systolic blood pressure. Body mass index was a substantial mediator of a negative indirect effect of socioeconomic levels on blood pressure in both genders. In a national study conducted in South Africa in 1998, women were found to be more aware, treated, and controlled for hypertension (51%, 36%, and 18%, respectively) than men (26%, 21%, and 10%), with ethnicity suggesting a modifying influence that was not statistically significant [70,71]. Higher SES, advanced age, greater interaction with primary care, and female sex have all been identified as positive drivers of awareness, treatment, and control in South Africa [67]. Individuals who reported being unable to read or write, those from lower-income homes, and immigrants were less likely to be aware of their condition, according to the researchers.

In rural South Africa, immigrants seem to be particularly vulnerable to hypertension. Jardim et al. [1] noted this gap and recommended migrants’ inclusion in South Africa’s health policy, particularly with regard to hypertension and its control.

### 4.5. Healthcare System

The health system is important for the awareness and control of hypertension disease. On the African continent, deficiencies in the healthcare system, patient adherence to medication, and physician inertia all play a part [66]. As observed in certain research, the limited availability of antihypertensive medicine and distance to health facilities are prevalent aspects of health systems in these locations, posing a threat to BP control [72,73]. A lack of time and competing priorities, on the other hand, are common patient and clinician factors that exacerbate the problem [74].

In South Africa, migrants face additional challenges in accessing healthcare due to language barriers, lack of valid immigration documentation, lack of health insurance, unfamiliarity with the healthcare system, and xenophobia [23,74]. A study conducted in Cape Town found that cross-border migrants experienced difficulties in communicating with healthcare providers due to language barriers, leading to misdiagnosis, delayed treatment, and poor health outcomes [75]. Furthermore, migrant women in South Africa face structural violence and experience poor healthcare infrastructure [23]. For example, the healthcare system’s response to the needs of migrant women in the Agincourt Health and Socio-Demographic Surveillance Site in South Africa is concerning. Research has shown that migrant women are less likely to access healthcare services compared to non-migrant women. At baseline, only 71.0% of migrant women had accessed healthcare services in the past year, compared to 33.1% of migrant men. The COVID-19 pandemic has further exacerbated these disparities, with a decline in healthcare service use among both migrant and non-migrant populations [54]. According to Pheiffer et al. [59], fewer migrants have accessed healthcare in the past year compared with non-migrants (50.6% vs. 56.0%).

The mental health of migrants is also a concern, as they often experience stress, anxiety, and depression due to their migration experiences [76]. Studies conducted in South Africa have shown that internal migration can have a significant impact on mental health. For instance, a study by Ginsburg et al. [43] found that internal migrants in South Africa reported lower levels of depressive symptoms compared to non-migrants. Munezero and Tomita [12] investigated hypertension and mental health challenges among 178 female African refugees in Durban, South Africa; their study identified exposure to adverse childhood experiences and depression as significant factors associated with hypertension. Another study by Thabana and Grace [77] revealed that refugees in Durban, South Africa, experienced high levels of mental health disorders, including depression and anxiety. Furthermore, a longitudinal study by Zinhle [19] found that rural–rural migrants in South Africa were at a higher risk of developing non-communicable diseases, including hypertension and diabetes, which can have a significant impact on mental health. A study conducted among African Caribbean adults with non-communicable diseases and mental health disorders also highlighted the importance of self-management in preventing disease progression [78]. Another study on African immigrants found that acculturation and length of stay in the host country were associated with cardiovascular risk screening, including hypertension, diabetes, and dyslipidemia [22].

However, hypertension awareness, treatment, and control have only been assessed on a rare basis in South Africa, and the migrant population has been significantly ignored [1]. Untreated cases of hypertension may likely affect the well-being of migrants [12], necessitating the World Hypertension League initiating a call for action to improve awareness, treatment, and control of hypertension in Africa, including increasing access to healthcare services, promoting healthy lifestyles, and reducing health disparities [78].

## 5. Conclusions

Given that South Africa is home to at least 2.9 million migrants, access to healthcare for the migrant community remains critical. In addition, migration is linked to acculturative stress; acculturation can contribute to hypertension due to the changes that occur during the process [1]. To ensure that migrants’ well-being is maintained and promoted, immigration policies on health must be updated and reviewed regularly.

The high burden of hypertension among African migrants in South Africa requires the implementation of several strategies. First, salt reduction policies should be extensively implemented to reduce dietary sodium intake, which is a major risk factor for hypertension [78]. For example, the South African government can enforce regulations on food manufacturers to reduce sodium content in processed foods. Public awareness campaigns can be launched to educate migrants about the risks of high sodium intake and promote healthier dietary habits.

The World Health Organization’s (WHO) HEARTS technical package should be implemented, which includes modules on healthy lifestyle, evidence-based treatment protocols, access to essential medicines and technology, risk-based cardiovascular disease management, team-based care, and systems for monitoring [78]. This can be achieved through collaboration between the South African Department of Health and non-governmental organizations (NGOs) that provide healthcare services to migrant communities.

Cost-effective screening and simplified treatment protocols should also be implemented to overcome treatment inertia by physicians and poor patient adherence to prescribed treatment [77]. For example, community-based health workers can be trained to conduct blood pressure screenings and provide basic treatment and referrals to migrant communities.

The decentralization of hypertension care to primary healthcare systems and communities can also increase access to care, within a context of integrated care [78]. This can be achieved through the establishment of community-based hypertension management programs that provide comprehensive care, including diagnosis, treatment, and follow-ups. Lastly, health-promoting environments should be encouraged through a sugar and alcohol tax and engagement with communities to promote healthy lifestyles [78]. This can be achieved through partnerships between government agencies, NGOs, and community organizations to promote healthy behaviors and provide support for migrants to adopt healthier lifestyles. South Africa can reduce the burden of hypertension among African migrants and promote their overall well-being by implementing these strategies. Additionally, addressing the social determinants of health, such as poverty, education, and employment, is crucial to reducing health inequities among migrant populations.

Future research should focus on evaluating the effectiveness of these strategies and identifying innovative solutions to address the complex health needs of migrant communities. Future research, such as longitudinal studies to track health outcomes among migrants over time or qualitative studies exploring personal experiences with hypertension management, and studies exploring cultural factors influencing health behaviors among African migrants, are suggested.

## Data Availability

No new data were created or analyzed in this study. Data sharing is not applicable to this article.
